# The Energetic Roots of Mental Illness: Mitochondrial Dysfunction in Psychiatry

**DOI:** 10.1192/j.eurpsy.2026.11623

**Published:** 2026-07-31

**Authors:** M. Arts, M. Zeilstra, K. M. Zeilstra-Kalt, L. D. Jonge

**Affiliations:** 1 Geriatric Psychiatry and Neuropsychiatry; 2 Lifestyle Medicine, GGZ WNB, Halsteren; 3 Integrative Medicine, Center de Wetering, Leeuwarden; 4 Integrative Medicine, Leonardo Scientific Research Institute, Groningen, Netherlands

## Abstract

**Introduction:**

Psychosocial stress and early-life adversity (ELA) increase lifetime risk for psychiatric and medical morbidity. Mitochondria, key organelles for energy, signaling, and steroidogenesis, are tightly coupled to stress mediators (glucocorticoids, catecholamines, cytokines) and are abundant in the brain. Acute stress adapts mitochondrial dynamics; chronic stress induces dysfunction with oxidative stress, altered mitochondrial DNA (mtDNA), and impaired bioenergetics, potentially embedding adversity biologically and shaping mood and cognition.

**Objectives:**

To synthesize evidence on (a) mitochondrial mechanisms in stress responding and allostasis, (b) associations between mitochondrial biomarkers and psychopathology, and (c) implications for prevention and treatment.

**Methods:**

Selective literature review integrating human and animal studies across neuroscience, endocrinology, and immunology. Focus on endocrine, inflammatory, and epigenetic pathways; neuroimaging; and peripheral biomarkers (mtDNA copy number, cell-free mtDNA, mitochondrial health index).

**Results:**

- Stress activates SAM/HPA axes and immune signaling, increasing energetic demand and ROS; prolonged exposure fragments mitochondria and damages mtDNA.
- Mitokines (ROS, calcium, cell free mtDNA) signal mitochondrial distress and promote inflammation; cell free mtDNA rises after psychosocial stress.
- ELA and lifetime psychopathology correlate with higher leukocyte mtDNA copy number and shorter telomeres in several cohorts; findings vary by tissue, chronicity, and medication.
- Animal models show that corticosterone or stress paradigms impair mitochondrial function and induce anxiety/depression-like behavior; brain studies implicate cortico-limbic circuits.
- Mitochondrial dysfunction occurs across disorders (major depressive disorder, anxiety, PTSD, bipolar disorder, schizophrenia, autism, neurodegeneration) with altered ETC activity and metabolism.
- Measurement challenges (cell heterogeneity, frozen samples, assay variability) limit comparability.

**Conclusions:**

Mitochondria lie at the nexus of stress biology and psychiatric outcomes, linking endocrine, immune, and metabolic pathways to neural circuits and behavior. Evidence supports a contributory role of mitochondrial dysfunction and stress-related “mitochondrial allostatic load.” Standardized, longitudinal studies with functional assays are needed to resolve heterogeneity, clarify reversibility, and test targeted interventions (e.g., anti-oxidative, metabolic, psychotherapeutic) as adjuncts to improve mental health.

**Disclosure of Interest:**

None Declared

